# Case Report: Shared manifestation, distinct etiologies: severe pulmonary hypertension in both mother and neonate

**DOI:** 10.3389/fped.2025.1612410

**Published:** 2025-08-26

**Authors:** Sihan Wang, Ping Yang, Hongmin Xi, Xiangyun Yin, Lili Ma, Liangliang Li, Xianghong Li

**Affiliations:** ^1^ College of Medicine, Qingdao University, Qingdao, China; ^2^ Neonatology Department, The Affiliated Hospital of Qingdao University, Qingdao, China

**Keywords:** pulmonary hypertension, hereditary hemorrhagic telangiectasia, *ACVRL1* gene, premature neonate, case report

## Abstract

**Background:**

Hereditary hemorrhagic telangiectasia (HHT) is a rare genetic disease. The prevalence of pulmonary arterial hypertension (PAH) in HHT patients is less than 1%. Severe pulmonary hypertension (PH) in pregnant woman due to HHT and reversible pulmonary hypertension in her neonate is even rarer.

**Methods:**

Cases of mother and newborn with PH are presented, including their clinical manifestations, diagnosis, and treatment. Additionally, literatures were reviewed to explore the various causes of the disease.

**Results:**

The mother was diagnosed with HHT caused by an *ACVRL1* gene variant, with her PH occurring as a complication of HHT type 2 (HHT2). The neonate was confirmed to be free of the *ACVRL1* gene variant, and her PH resulted from impaired placental perfusion and adverse intrauterine environment secondary to severe maternal PH and anemia.

**Conclusion:**

Attention should be paid to the progression of the disease and the comprehensive management strategies during pregnant HHT patient. Moreover, neonates born to HHT-affected mothers require evaluation for both HHT-related PH and reversible PH secondary to adverse intrauterine factors. This report aims to enhance the recognition of familial manifestations of HHT and raise awareness of the occurrence of postnatal PH in neonates of mothers with HHT, thereby facilitating early detection and timely intervention.

## Introduction

Hereditary hemorrhagic telangiectasia (HHT) is a rare autosomal dominant disorder, the incidence is about 1–2/10 000 (1). It is characterized by recurrent epistaxis, cutaneous telangiectasia, PH, and arteriovenous malformations (AVMs) affecting the lungs, gastrointestinal tract, liver, and brain (2). Pregnancy poses significant risks for women with HHT, particularly concerning potential cardiopulmonary complications. This report focuses on one key complication of HHT—pulmonary hypertension (PH)—which significantly increases morbidity and mortality, especially during pregnancy. PH is characterized by elevated pulmonary arterial pressure resulting from various pathophysiological mechanisms, defined as a mean pulmonary artery pressure ≥20 mmHg measured by right heart catheterization at rest (3). In HHT patients, PH is associated with significantly elevated morbidity and mortality rates (1). This study aims to elucidate the relationship between HHT and PH, through a case report of maternofetal PH resulting from distinct etiologies, while highlighting the importance of identifying specific PH susceptibility groups.

### Case description

A 37-year-old pregnant woman presented with a 20-year history of epistaxis and allergic rhinitis for 8 years, without any other comorbidities. She had previously delivered two male infants, one of whom exhibited recurrent epistaxis. Her father also had a documented history of epistaxis. Symptomatic treatment was provided without further investigation. Before admission to our hospital, she received prenatal care at another institution. She was diagnosed with gestational diabetes mellitus at 24 weeks. During the current pregnancy, the patient developed anemia secondary to significant recurrent epistaxis. At 17 weeks pregnant, she was found to have a hemoglobin level of 98 g/L, without taking iron supplements regularly. Progressive anemia developed, and exertional chest tightness subsequently occurred at 27 weeks. One day prior to admission, blood tests at another hospital revealed a hemoglobin level of 63 g/L, accompanied by significant chest tightness, fatigue, and tinnitus. Echocardiogram revealed the pulmonary artery pressures at 87 mmHg, indicates severe PH. At 29^+2^ weeks, she presented to our hospital with pulmonary artery pressure of 109 mmHg and was admitted to the obstetrics department. Prior to delivery, her pulmonary artery pressure reached 122 mmHg. Following a multidisciplinary consultation at 29^+3^ weeks of gestation, the team recommended therapeutic termination of pregnancy, packed red blood cell transfusion to correct anemia, antenatal dexamethasone to promote fetal lung maturation, genetic testing to identify the cause, and oral sildenafil (25 mg three times daily) for pulmonary hypertension management. At 29^+^⁴ weeks, intravenous treprostinil (initiated at 1.25 ng/kg/min) via infusion pump and oral sildenafil (25 mg once daily) were initiated. After caesarean section at 29^+5^ weeks, subcutaneous treprostinil (initiated at 1.25 ng/kg/min) was administered for one mouth, supplemented with oral macitentan (10 mg once daily). The right heart catheterization and pulmonary arteriography of the mother revealed precapillary PH. Oral selexipag (0.2 mg twice daily) was added to the existing regimen, with subsequent dosage adjustments guided by pulmonary artery pressure. Maternal pulmonary artery pressure was 78 mmHg – reduced from pre-pregnancy levels but remained significantly higher than normal. Her symptoms of chest tightness and breathlessness had improved, with enhanced exercise tolerance.

The preterm neonate with a gestational age of 29^+5^ days was born weighing 990 grams and had Apgar scores of 10 at both 1 and 5 min after birth. There was no parental consanguinity. There were no signs of intrauterine distress or premature rupture of membranes, nor history of amniotic fluid contamination. However, she rapidly manifested with progressively worsening respiratory distress and developed respiratory distress syndrome and progressive hypoxic respiratory failure despite mechanical ventilation and two courses of pulmonary surfactant therapy. Echocardiography at 12 h after birth confirmed persistent pulmonary hypertension of the newborn (PPHN) with pulmonary artery pressures up to 55 mmHg and an oxygen index of 32, necessitating inhaled nitric oxide (iNO) therapy. Clinical improvement allowed for discontinuation of iNO therapy at 88 h, with PH resolution confirmed at 7 days. The neonate was discharged following a 72-day hospitalization, with subsequent outpatient follow-ups demonstrating no evidence of PH. By 9 months postpartum (corrected gestational age: 7 months), body weight increased from 2.47 kg (<3rd percentile) at discharge to 6.3 kg (10th–15th percentile), whereas body length increased from 45 cm (<3rd percentile) to 66 cm (20th percentile). Head circumference stabilized at 40 cm (3rd percentile). Both motor and language development were within normal ranges. Longitudinal monitoring of anthropometric parameters, neurodevelopmental milestones, and pulmonary artery pressure will be implemented.

**Figure 1:**
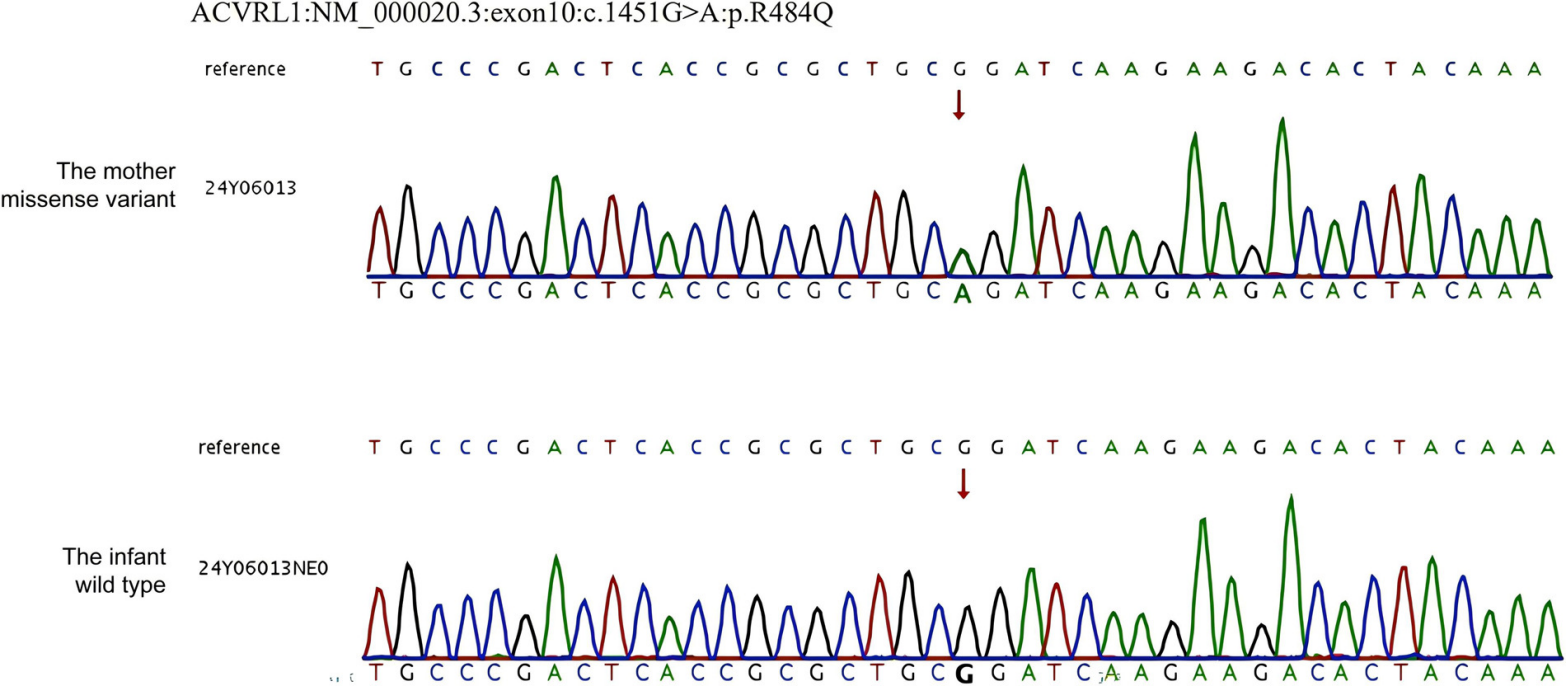
Sanger sequencing results of the mother and infant. WES revealed a pathogenetic variant c.1451G > A (p. R1478Q) of the *ACVRL1* gene (NM_000020.3).

**Figure 2:**
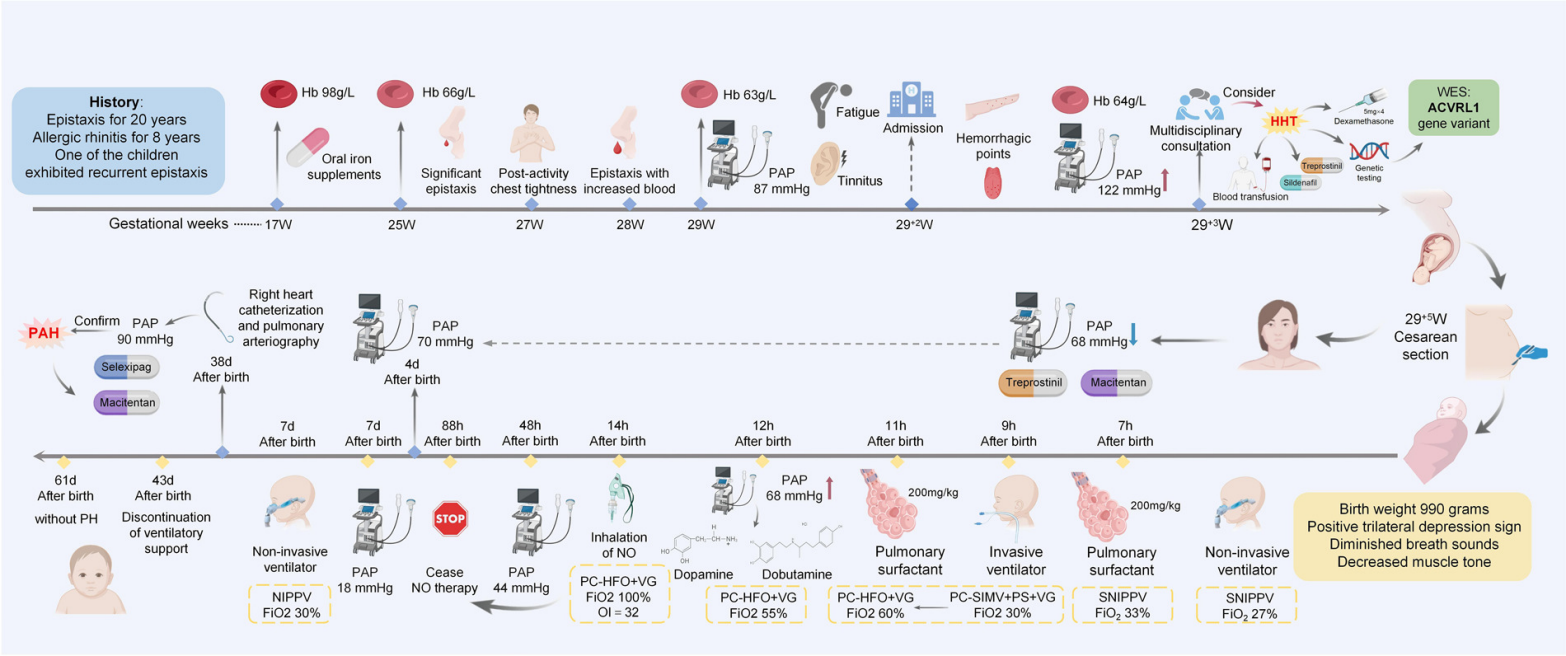
Timeline. It summarizes key clinical parameters including symptomatic manifestations, diagnostic evaluations, and therapeutic interventions for both the mother and the neonate. Hb, hemoglobin; PAP, pulmonary artery pressure; WES, whole-exome sequencing; NO, nitric oxide.

Whole-exome sequencing (WES) revealed a heterozygous missense mutation in the *ACVRL1* gene, c.1451G>A in the mother, which is known to cause HHT, consistent with autosomal dominant inheritance of HHT. No pathogenic ACVRL1 variants in this site were identified in the infant (Figure 1). The treatment flow chart is illustrated in Figure 2.

## Discussion

HHT arises from mutations in *ENG*, *ACVRL1*, *MADH4*, or *GDF2*, with *ENG* (encoding for the Endoglin protein) and *ACVRL1* (encoding for the ALK1 protein) mutations accounting for ∼85% of cases, all encoding components of the transforming growth factor beta (TGF-β) signaling pathway. TGF-β pathway proteins are critical endothelial regulators that orchestrate vascular endothelial cell proliferation, migration, and apoptosis, thereby maintaining vascular integrity. These genetic alterations impair angiogenesis and endothelial remodeling, resulting in fragile and structurally compromised vessels that manifest as telangiectasia and AVMs (1, 4). The ENG gene encodes endoglin, with loss-of-function mutations causing hereditary hemorrhagic telangiectasia type 1 (HHT1), whereas ACVRL1 encodes ALK1, whose pathogenic variants underlie HHT type 2 (HHT2). MADH4 variants cause juvenile polyposis/HHT overlap syndrome, while GDF2 encodes BMP9, which can bind to endoglin protein and ALK1 protein, leading to overlapping phenotypes of HHT1 and HHT2. Phenotypically, HHT1 demonstrates higher penetrance of pulmonary/cerebral AVMs and mucocutaneous telangiectasia, while HHT2 patients have an increased risk of hepatic and pulmonary AVMs and PH (5). Therefore, compared to HHT1 patients, HHT2 patients should pay more attention to monitoring pulmonary arterial hypertension.

PH represents a well-documented but uncommon complication of HHT, affecting ∼15% of patients (6). HHT-associated PH is classified into two main types: precapillary and postcapillary PH. Precapillary PH, also termed pulmonary arterial hypertension (PAH), is primarily characterized by vascular remodeling in small pulmonary arteries. This process is exacerbated by HHT-associated gene mutations, due to shared pathogenic mutations in the BMP9/ALK1/endoglin signaling pathway (7). Mutations in *ACVRL1* impair apoptotic signaling in vascular smooth muscle cells, resulting in enhanced cellular proliferation and pathological vascular remodeling, ultimately causes PAH (4). The prevalence of PAH in HHT patients is estimated to be less than 1% (8). In a cohort study of 2,598 HHT patients, right heart catheterization identified PH in 38 cases, with only 3 meeting PAH criteria, further confirming its rarity (9). Postcapillary PH is predominantly associated with systemic AVMs and high-output cardiac states (1, 10). Notably, HHT can be complicated by hereditary pulmonary hypertension (HPAH), mediated by pathogenic variants in *ACVLR1*, *ENG*, *SMAD4*, and *BMP9* genes. However, the co-occurrence of HPAH and HHT remains rare (4, 11).

For the treatment of HHT, current treatment focuses on symptom control, and drug targets for the mechanism of action of the disease are still under further study. Emerging therapeutic candidates include tacrolimus, bevacizumab, octreotide, and thalidomide (4). Due to the rarity of HHT and PAH, there are currently no randomized controlled trials to aid in guiding specific treatments for HHT-PAH. It is usually managed with guideline-recommended therapies for idiopathic PAH, including endothelin receptor antagonists (e.g., ambrisentan, bosentan, macitentan), soluble guanylate cyclase stimulators (e.g., riociguat), phosphodiesterase inhibitors (e.g., sildenafil, tadalafil, vardenafil), and prostanoids (e.g., epoprostenol, treprostinil, iloprost, and selexipag) (1). During the mother's PAH treatment, the following medications were used in combination. Treprostinil binds to prostacyclin receptors on smooth muscle cell, leading to increased cyclic adenosine monophosphate and reduced intracellular calcium. Sildenafil relaxes smooth muscle cells by increasing cyclic guanosine monophosphate levels, and by that pathway also reduces intracellular calcium. Macitentan promotes vasodilation by reducing endothelin-1 binding to A and B type endothelin receptors, thereby lowering morbidity and mortality and improving exercise capacity. Selexipag is a prostacyclin receptor agonist, and exhibits a selectively high affinity for prostacyclin receptors (12–14). However, all of the endothelin receptor antagonists and riociguat are considered teratogenic in animal experiments, so their use is contraindicated during pregnancy (15). Therefore, during her pregnancy, sildenafil and treprostinil were initially used in combination. After delivery, medications were adjusted based on the mother's pulmonary artery pressure levels. The aim was to enhance pulmonary vasodilation by combining agents with different mechanisms of action. However, no clinical trial or animal study data are currently available regarding the interactions and efficacy of these specific combinations in this case. However, PAH-specific therapies may exacerbate HHT-related complications through distinct mechanisms. Eric et al. demonstrated that prostanoids (e.g., epoprostenol) improve hemodynamics in HHT-PAH patients but concurrently increase bleeding risk via antiplatelet effects, particularly problematic in HHT populations with inherent vascular fragility (6). Tomás et al. reported that endothelin receptor antagonists (e.g., macitentan) reduce PAH-related mortality but increases the incidence of anemia (16). In this case, long-term dual therapy with prostanoids and macitentan during the postpartum period required vigilant monitoring for hemoglobin decline and epistaxis frequency, necessitating dose adjustments guided by multidisciplinary consensus.

Histopathological findings in HHT-associated PAH are similar to those observed in idiopathic PAH (10). Given the woman's severe phenotype, this condition is closely associated with her pregnancy. The severe PH during pregnancy may be attributed to the following factors: (1) A >50% increase in circulating blood volume during pregnancy imposes hemodynamic stress on the already vulnerable vascular system in HHT patients, potentially promoting PAH development. (2) Elevated estrogen and progesterone levels during pregnancy may promote PH by affecting vascular endothelial cell growth and migration, potentially via modulation of downstream TGF-β signaling molecules; however, the specific mechanisms in ACVRL1 mutations require further investigation. (3) Severe anemia, reflecting reduced hemoglobin levels, decreases oxygen transport capacity, potentially causing cardiovascular pathological changes. Thickening of the intima and adventitia of the pulmonary arteries, along with a decrease in the cross-sectional area of the pulmonary vessels, results in elevated pH (17). (4) While pregnancy induces a hypercoagulable state that could cause PAH via chronic thromboembolic pulmonary hypertension (18), right heart catheterization and pulmonary angiography revealed no significant filling defects, stenosis, or occlusive lesions, excluding this factor. The patient exhibited only epistaxis and fatigue pre-pregnancy but developed pulmonary arterial hypertension during the third trimester. We considered that pregnancy and anemia caused by pregnancy-aggravated recurrent epistaxis represent significant risk factors for exacerbating HHT-related complications. However, persistent PH following pregnancy termination in the mother indicates that HHT represents the primary etiology. Her recorded pulmonary artery pressure of 122 mmHg indicates severe PH, historically associated with a mortality rate of 56%, which is still 11.5% with contemporary treatment strategies (19). Studies demonstrate that HHT2 with comorbid PAH exhibits faster disease progression and poor clinical prognosis compared to other HHT subtypes (4, 8).This may also explain the severe manifestation of the maternal phenotype.

The premature neonate developed PPHN soon after birth despite the absence of the *ACVRL1* gene variant by genetic testing to rule out the diagnosis of HHT and HPAH (Figure 1). The classic pathogenesis is considered as impaired placental perfusion and adverse intrauterine environment secondary to severe maternal PH and anemia. This pathophysiology involves impaired postnatal circulatory transition, which is characterized by persistent elevation of pulmonary vascular resistance (PVR). Under physiological conditions, fetal circulation maintains high PVR with oxygenation dependent on placental exchange, transitioning postnatally to low-resistance pulmonary circulation. Dysregulation of the normal postnatal circulatory transition — systemic vascular resistance (SVR) elevation and acute PVR reduction mediated by pulmonary vasodilation — results in PPHN (20, 21). Impaired placental perfusion can lead to tissue hypoxia during fetal development. In pulmonary artery smooth muscle, hypoxia inhibits ROS production, thereby suppressing O2-sensitive voltage-gated potassium (Kv) channels, causing membrane depolarization, which activates voltage-gated calcium channels (CaL), increases [Ca2+]i, induces pulmonary vasoconstriction, elevates PVR, and results in PPHN (22). Additionally, complex interplay of angiogenic imbalance, inflammation, oxidative stress, epigenetic alterations and elevated endoplasmic reticulum stress may also be associated with the formation of PPHN [26].

Additionally, prenatal use of sildenafil to reduce pulmonary artery pressure in pregnant women may have adverse effects on the fetus. Consistently, a Dutch group had previously demonstrated this phenomenon. The randomized controlled trial investigating prenatal sildenafil use among pregnant women (*N* = 216; 108 assigned to sildenafil and 108 to placebo) demonstrated a significantly higher incidence of neonatal pulmonary hypertension in the sildenafil group (18.8%) vs. the placebo group (5.1%). Sildenafil can cross the placenta to reach fetal tissues, exhibiting biological activity within the fetal pulmonary vasculature that promotes pulmonary vasodilation and placental vascular dilation (23, 24).The Dutch group hypothesized that abrupt postnatal cessation of sildenafil may result in a “rebound” vasoconstriction or lack of further vasodilation, compromising the neonate's transition to extrauterine circulation (25, 26). Collectively, the underlying mechanism still remains speculative and warrants further clinical trials and animal experiments under the HHT model.

Although several HHT-PAH cases have been reported, this represents the first documented case of a mother with HHT-PAH complicated by neonatal PPHN and reveals a novel mutation site associated with HHT. Study limitations include the absence of genetic testing in other family members due to their preference, and validation limited to the ACVRL1 gene in the neonate. Longitudinal follow-up is planned for the proband, with additional genetic testing (e.g., for ENG, MADH4, and GDF2) to be performed if pulmonary hypertension symptoms manifest. Early molecular diagnosis is necessary to confirm the diagnosis to strengthen proactive surveillance for HHT-associated complications and managed as early as possible to improve the prognosis.

The rare case of HHT with precapillary PH during pregnancy aims to enhance clinical recognition of HHT and emphasize the importance of echocardiogram screening for early detection of potential complications in pregnant HHT patients. Comprehensive management strategies should include genetic counseling, prenatal diagnosis, and multidisciplinary treatment planning. The occurrence of PH in the neonate suggests that in addition to considering HHT-related PH, reversible PH secondary to adverse intrauterine factors should also be taken into account. These case reports provide valuable insights into the HHT, potentially improving perinatal management strategies in HHT patients and increasing clinical attention for postnatal PH in neonates born to HHT-affected mothers.

## Data Availability

The datasets presented in this article are not readily available because of ethical and privacy restrictions. Requests to access the datasets should be directed to the corresponding authors.
